# Southern Hemisphere initiation of the mid-Pleistocene transition

**DOI:** 10.1126/sciadv.aea6811

**Published:** 2026-03-04

**Authors:** Chandranath Basak, A. K. Isuri U. Kapuge, Jesse R. Farmer, Emily Symes, Jennifer L. Middleton, Julia Gottschalk, Helge W. Arz, Frank Lamy, Gisela Winckler, Anna P. S. Cruz

**Affiliations:** ^1^ Department of Earth Sciences, University of Delaware, Newark, DE 19716, USA.; ^2^ School for the Environment, University of Massachusetts Boston, Boston, MA 02125, USA.; ^3^ Lamont-Doherty Earth Observatory of Columbia University, Palisades, NY 10964, USA.; ^4^ Institute for Geosciences, Kiel University, Kiel 24118, Germany.; ^5^ Leibniz Institute for Baltic Sea Research Warnemünde, Rostock 18119, Germany.; ^6^ Alfred Wegener Institute, Helmholtz-Center for Polar and Marine Research, Bremerhaven 27570, Germany.; ^7^ MARUM–Center for Marine Environmental Sciences, Bremen 28359, Germany.; ^8^ Columbia Climate School, Columbia University, New York, NY 10025, USA.

## Abstract

The mid-Pleistocene transition [MPT; between ~1.25 and 0.7 million years ago (Ma)] marks when Earth’s glacial pacing switched from 41,000- to 100,000-year cyclicity. Here, we present a South Pacific hydrogenous neodymium isotope record tracking changes in deep ocean circulation spanning 1.5 to 0.5 Ma. This record indicates that the MPT was not triggered by Northern Hemisphere (NH) processes. Instead, the growth of Antarctic ice sheets to a critical size (~1.8 Ma) primed the climate system for the MPT, with iceberg release and melting transferring buoyancy out of the Southern Ocean. This iceberg-associated buoyancy redistribution ultimately reduced deep water formation in the NH, an observation supported by our data. Once the MPT was initiated in the Southern Hemisphere, its subsequent climatic changes were dominated by NH processes.

## Teaser

A South Pacific neodymium isotope time series suggests Antarctic ice sheet growth triggered the mid-Pleistocene transition.

## INTRODUCTION

The Pleistocene Epoch [2.58 to 0.01 million years ago (Ma)] is the most recent interval when the Earth experienced cyclic glaciations with alternating cold (glacial) and warm (interglacial) climates, as diagnosed through benthic foraminiferal oxygen isotope compilations (*1*). While early Pleistocene glaciations occurred at a characteristic oscillation of ~41,000 years (41 kyr), the late Pleistocene glaciations had a periodicity of ~100 kyr as ice sheets became more voluminous. This transition from 41 to 100 kyr glacial cycles happened between ~1.25 to 0.7 Ma, an interval commonly recognized as the mid-Pleistocene transition (MPT) (*2*, *3*). No noteworthy variations in Earth’s orbital configuration occurred across the MPT that could explain the change in glacial periodicity. Instead, Earth’s internal nonlinear climate feedbacks are often considered to have brought about the changes (*1*, *4*–*10*).

Among the prevailing hypotheses, one that has garnered considerable attention is that a major disruption of the Atlantic Meridional Overturning Circulation (AMOC) occurred between 0.95 and 0.86 Ma ago, which drove an atmospheric CO_2_ drawdown that led to the growth of high-latitude ice sheets, and ultimately, the first appearance of the 100 kyr glacial cycle (*6*). However, a recent highly resolved Nd isotope record from the South Atlantic exhibited no prominent change in deep ocean circulation across the MPT (*11*). These evolving views on MPT deep ocean circulation are primarily focused on the sequence of events occurring around 0.9 Ma in the deep Atlantic Ocean.

While changes in deep ocean circulation at and around 0.9 Ma have been cited as pivotal to the MPT (*6*–*8*), here, we report a high-resolution deep ocean circulation reconstruction from the Southern Ocean that shows a preceding, pervasive circulation change starting at 1.4 Ma and culminating ~1.25 Ma. On the basis of this evidence, we propose that the MPT was initiated by a Southern Hemisphere–driven reorganization of deep ocean circulation, linked to the Antarctic ice sheet (AIS)’s expansion into the marine realm. In addition, atmosphere-ocean carbon exchange across the Southern Ocean and carbon storage in Southern Ocean–sourced deep waters are considered proximal contributors to climate changes across both the MPT and late Pleistocene glacial cycles (*3*, *6*, *7*, *9*, *11*). As such, constraining deep ocean circulation changes in the Southern Ocean is critical for a holistic understanding of the MPT and an evaluation of the mechanisms leading to its occurrence.

## RESULTS

### Core site and approach

**Fig. 1.**
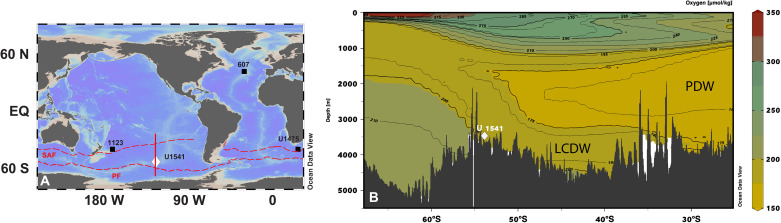
Core sites and water mass structure in the central South Pacific. (**A**) Map showing site locations of Site U1541 (white diamond) and other records (filled black squares) in the South Pacific and Atlantic Ocean. Broken red lines are polar front (PF) and sub-Antarctic front (SAF). Solid red line demarcates along which the section plot is drawn. (**B**) Section plot showing dissolved oxygen in the central South Pacific. In the modern ocean, Site U1541 is bathed by Lower Circumpolar Deep Water (LCDW). Low-oxygen water mass is Pacific deep water (PDW). All water masses shoal toward the surface near Antarctica along tilted isopycnals.

We report a high-resolution (median sampling resolution ~1 sample/8 kyr) hydrogenous (i.e., representing seawater) Nd isotope time series from IODP Site U1541 in the central South Pacific (54°13′S, 125°25′W, water depth 3604 m; Fig. 1A). The Nd isotope (expressed as ε_Nd_, fig. S1) record spanning 1.5 to 0.5 Ma is interpreted as a tracer of water mass mixing. This interpretive framework is based on linear mixing between two endmembers, namely unradiogenic Northern Component Water [NCW; ε_Nd_ = −10 to −14 (*12*, *13*)] and radiogenic Pacific Deep Water [PDW; ε_Nd_ = −3 to −4 (*14*)]. Globally, in open ocean, high/low values in ε_Nd_ records that lie along AMOC flow path have been classically interpreted as a smaller/larger proportion of North Atlantic influence, respectively. Therefore, deep-ocean ε_Nd_ records may be explained by changes in deep ocean circulation driven by proportions of constituent water masses alone, with no concomitant changes in the endmember composition. Nonconservative sources of Nd are unlikely to be the dominant driver at Site U1541 and hence we interpret water mass mixing to dominate our ε_Nd_ record (Supplementary Materials). Modern hydrography indicates that the location of Site U1541 is bathed by Lower Circumpolar Deep Water (LCDW) (Fig. 1B and fig. S2). The LCDW in the South Pacific represents a modified remnant of saline North Atlantic Deep Water (NADW) mixed with PDW, a low oxygen water mass that occupies depths between 1500 and 3500 m today (*15*). Therefore, Site U1541 is well positioned to capture deep ocean circulation changes across the MPT expressed as relative proportions of Atlantic- and Pacific-sourced water masses in the South Pacific basin.

### Neodymium isotope time series

**Fig. 2.**
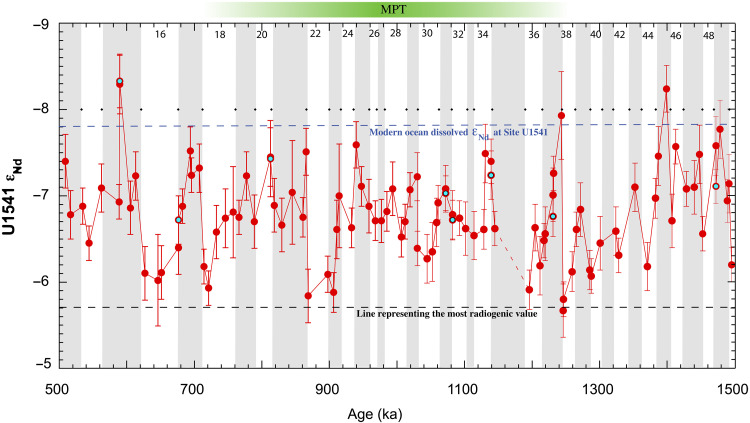
ε_Nd_ time series for Site U1541 spanning 0.5 to 1.5 Ma. The blue and black broken lines represent the modern ocean dissolved Nd isotope and most radiogenic value in the time series respectively. The black diamonds represent the boundary of marine isotope stages (*1*). The vertical gray bars and the numerals are the interglacial and glacial periods respectively. Core gap is shown as broken line. Measurement uncertainties (2SD) are shown by vertical errors bars. Filled cyan symbols are replicate analyses.

Between 1.5 and 0.5 Ma, the ε_Nd_ values at Site U1541 oscillated between −5.7 and −8.3 with semiregular cyclicity (Fig. 2). Glacial periods show higher ε_Nd_ values than interglacial periods, a feature shared with previous ε_Nd_ reconstructions from the Atlantic Ocean (fig. S3). Beyond these large-scale glacial-interglacial fluctuations, the pattern of ε_Nd_ changes among consecutive glacial and interglacial periods varied across three intervals: the pre-MPT (1.5 to 1.25 Ma), MPT (1.25 to 0.7 Ma), and post-MPT (0.7 to 0.5 Ma) (Fig. 2). During the pre-MPT interval, interglacial ε_Nd_ values either reached or were lower than the modern-day dissolved ε_Nd_ value of −7.6 (*16*). Beginning around 1.4 Ma, a general trend emerged of increasing ε_Nd_ with each consecutive glacial cycle, peaking at the least negative (most radiogenic) value of −5.67 at marine isotope stage (MIS) 38 (~1.25 Ma).

The difference between pre-MPT median glacial (−6.52) and interglacial (−7.46) ε_Nd_ values is approximately 1 epsilon unit. During the MPT, Site U1541 ε_Nd_ values continued to fluctuate between glacial and interglacial periods, but the magnitude of change between median glacial (−7.05) and interglacial (−6.71) values was reduced (~0.34 epsilon units). A second notable radiogenic excursion occurred during MIS 22 (0.9 Ma), when Site U1541 ε_Nd_ reached −5.88. In the post-MPT period, ε_Nd_ values continued to oscillate in the same glacial-interglacial pattern, with a ~1 epsilon unit change between median glacial (−7.23) and interglacial (−6.28) values, similar to the pre-MPT (Fig. 2). The pre- and post-MPT glacial and interglacial median ε_Nd_ values are similar (*P* > 0.05 at 95% confidence interval), suggesting no step change in the proportions of NCW or PDW at Site U1541 across the MPT (*11*). However, despite the lack of large shifts in deep ocean circulation across the MPT, the notable ε_Nd_ excursions, particularly the one predating MIS 22, requires explanation. We next evaluate the role of Southern Hemisphere in setting up the boundary conditions leading up to the MPT.

## DISCUSSION

### AMOC disruptions in the central South Pacific deep circulation

**Fig. 3.**
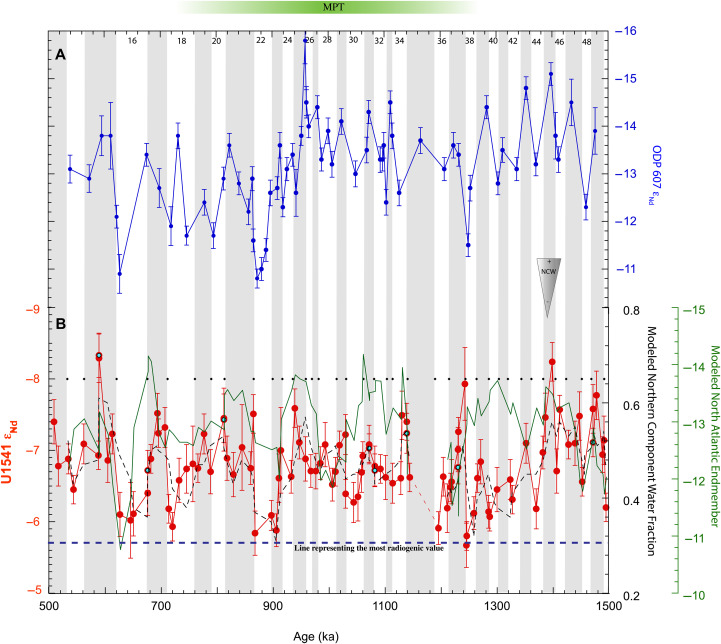
Comparison between ε_Nd_ time series from Site 607 and Site U1541. (**A**) North Atlantic hydrogenous ε_Nd_ time series (*43*), representing changes in Northern Component Waters (NCW) with time. (**B**) Central South Pacific hydrogenous ε_Nd_ time series (filled red circles). The error bars are external reproducibility (2SD) of international standard JNdi-1 analyzed during analytical sessions. The cyan filled circles are samples to demonstrate reproducibility. The solid green line is the running mean of the modeled North Atlantic endmember through time. Broken black line is the running mean of the modeled fraction of Northern Component Water at Site U1541. Black dots indicating the boundary of marine isotope stages (MIS) according to (*1*). Core gap in Site U1541 is shown as broken red line between 1140 and 1190 ka. Broken blue line represents the most radiogenic value in Site U1541 ε_Nd_ record.

AMOC disruptions in ε_Nd_ records are identified as positive excursions, indicating a reduction in the presence of unradiogenic NCW, which is typically associated with a reduction or suppression of the production of unradiogenic deep waters in the North Atlantic (e.g., NADW). Yehudai *et al.* (*8*) presented evidence in favor of a Northern Hemisphere trigger of the MPT via weathering, resulting in exposed crystalline bedrock, leading to increased frictional surface and large ice sheet growth (*10*, *17*). They argued that between ~0.95 and 0.86 Ma, the deep Atlantic had less NCW indicating prominent changes in water mass structure, which they named the “MPT-AMOC disruption” (Fig. 3). Before this disruption, between 0.98 and 0.95 Ma, the North Atlantic ε_Nd_ record (Site 607, Fig. 1A, 41°N, 32°W; 3427 m) is unusually negative, suggestive of ice sheet weathering/erosion of old continental craton off Canada [ε_Nd_ < −30; (*18*)]. They further claimed that marine terminating Northern Hemisphere ice sheets produced freshwater melt, which weakened the deep circulation cell in the North Atlantic and produced the disruption (~0.9 Ma, MIS 22). The disruption, once produced, facilitated atmospheric CO_2_ drawdown via reduced exchange between Antarctic surface and deep waters (*19*), resulting in cooling and high latitude ice-sheet growth that stabilized the 100 kyr glacial cycle.

Given the above argument, the 0.9 Ma radiogenic ε_Nd_ excursion (during MIS 22) should be globally present and unique. Available data show that a radiogenic ε_Nd_ excursion at MIS 22 is indeed present in most records but not unique as previously suggested (fig. S3). In the central South Pacific, the Site U1541 ε_Nd_ record exhibits two glacial periods with elevated ε_Nd_ values: MIS 38 (~1.25 Ma, ε_Nd_ −5.80 ± 0.20) and MIS 22 (~0.9 Ma, ε_Nd_ −5.84 ± 0.23) (Fig. 2). The ε_Nd_ values of both periods are within measurement error of each other and are robustly defined by multiple data points. Using the interquartile range method, peak ε_Nd_ values during MIS 22 and 38 are outliers compared to other glacial ε_Nd_ values in the Site U1541 record, making these glacials statistically distinct from the other glacial intervals. Without invoking any changes in the AMOC, these radiogenic ε_Nd_ excursions could be explained via a greater production of deep water in the Pacific carrying a radiogenic ε_Nd_ signature, suppressing the unradiogenic NCW signal at U1541. However, carbonate mass accumulation rates in the North Pacific (*20*) indicate no marked changes to support higher PDW formation across the MPT or immediately after. Therefore, we suggest that the radiogenic ε_Nd_ excursions at MIS 22 and 38 in the South Pacific are both linked to AMOC changes, although the mechanisms driving AMOC during these excursions may not be similar.

### AMOC versus endmember change

Since the inception of the ε_Nd_ as a tracer of water masses, linear mixing models with fixed endmember Nd isotopic compositions have been implemented. Evidence in favor of stable endmembers for the last 2 Ma comes from a compilation of low resolution Fe-Mn crust records from the Pacific and the North Atlantic (*6*). However, a recent study covering the last 100 kyr suggest that changes in the ε_Nd_ endmember in the North Atlantic could explain North Atlantic ε_Nd_ records without any changes to water mass mixing proportions (*21*). Growth and decay of ice sheets are known to erode basement rocks with different ε_Nd_ values due to change in provenance and/or type of weathering (*8*, *22*, *23*); therefore, the possibility of North Atlantic endmember change cannot be ruled out.

To address this, we developed a two endmember linear mixing model with dynamic endmember ε_Nd_ compositions (Supplementary Materials). The model results show a considerable variation in North Atlantic endmember ε_Nd_ composition over time (Fig. 3B), supporting previous propositions of a variable northern endmember (Fig. 3B) (*21*–*23*). The modeled Pacific endmember showed a narrow range of ε_Nd_ values (Supplementary Materials). This stability is expected; PDW is a recycled/upwelled deep water sourced from the south (*15*) and largely attains its ε_Nd_ via interaction with volcanic rocks along the margins and perhaps the reactive seafloor (*24*). Since PDW does not form by sinking of surface water, surface processes such as ice-sheet–related weathering inputs are not expected to directly alter the ε_Nd_ of PDW.

The cyclic pattern of the modeled North Atlantic endmember ε_Nd_ suggests a climatic control via expansion and contraction of the Northern Hemisphere ice sheets and their resulting weathering input. Glacial weathering preferentially releases Nd from minerals with lower Sm/Nd than the bulk soil or rock (*25*), resulting in lower ε_Nd_. Therefore, weathering during ice inception and expansion would result in lower ε_Nd_ NCW endmember values and opposite for ice contraction. The MPT and post-MPT periods show low and high North Atlantic ε_Nd_ endmember compositions coinciding with glacial and interglacial climates, respectively. Notably, the overall similarity in the pattern of changes in the modeled North Atlantic ε_Nd_ endmember and U1541 ε_Nd_ is strong starting after 1.25 Ma (Fig. 3B). The variation in modeled fraction of NCW also agrees with the Site U1541 ε_Nd_, conforming to the idea of less influence of NCW during glacials in comparison to a higher NCW proportion during interglacials (Fig. 3B). We infer from this that the climate driver (i.e., ice growth and decay) that controlled the North Atlantic endmember ε_Nd_ also controlled deep water formation in the North Atlantic, which eventually propagated to the study site. From this model output, we suggest that MPT and post-MPT hydrogenous Nd isotopes at Site U1541 integrate a combination of water mass mixing and changes in North Atlantic endmember composition.

However, a distinct pattern is observed during the pre-MPT period (MIS 46-38): While Site U1541 ε_Nd_ gradually increased along with a gradual decrease in modeled NCW contribution, the associated North Atlantic endmember did not follow a similar pattern and is represented by lower ε_Nd_ values across multiple glacial cycles (Fig. 3). Therefore, the decoupling between the North Atlantic ε_Nd_ endmember and deep water mixing proportion at Site U1541 is not driven by Northern Hemisphere weathering, but rather by changes in water mass mixing. Thus, although the Nd isotope excursions during MIS 38 and 22 are quantitatively similar, the underlying mechanism driving the changes during MIS 38 leading up to the MPT driven by deep-ocean mixing and not weathering of the Northern Hemisphere.

### Antarctic trigger of pre-MPT AMOC weakening

**Fig. 4.**
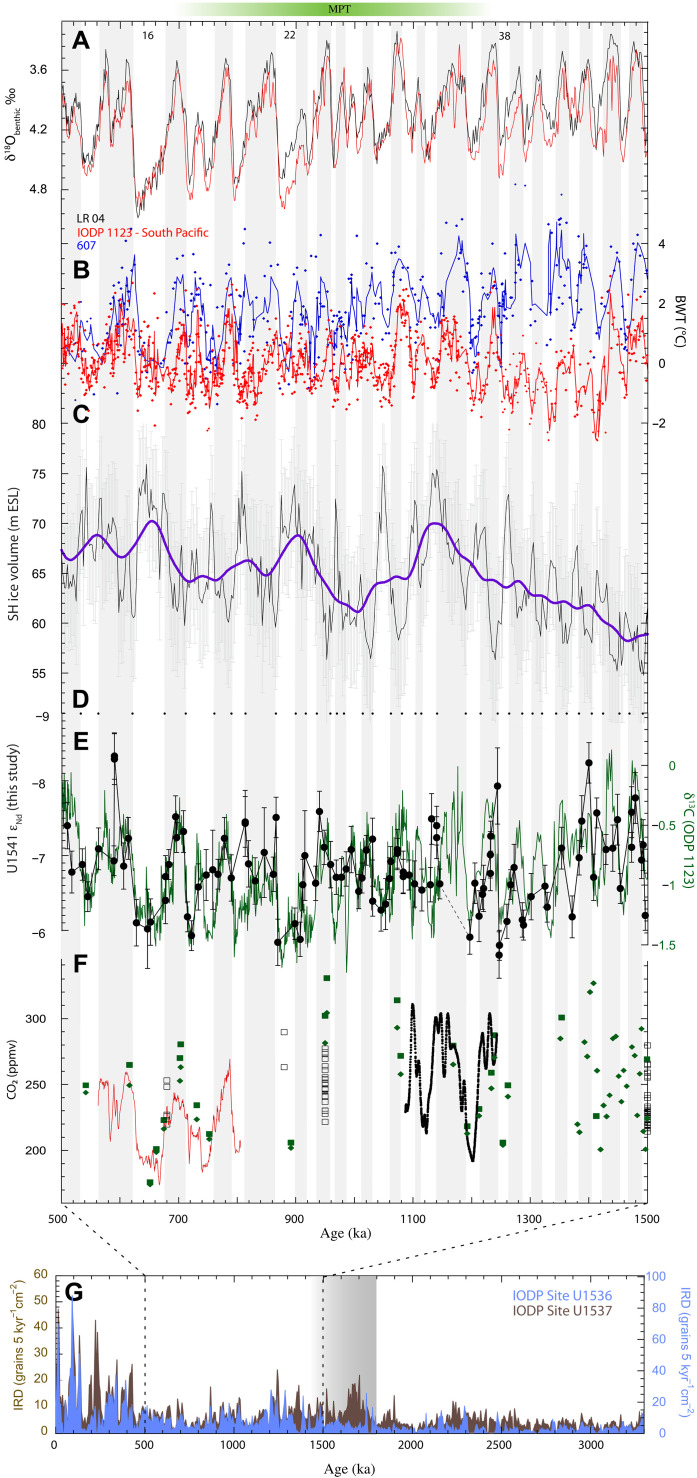
Proxy compilation showing climate records in the context of Site U1541 ε_Nd_ time-series. (**A**) Comparison of benthic δ^18^O between LRO4 global stack (*1*) and ODP Site 1123 off Chatham Rise (*5*). (**B**) Bottom water temperature (BWT) derived from foraminiferal Mg/Ca from ODP Site 607 (*26*, *27*) and Site 1123 (*5*). Blue and red lines are four-point running mean of plotted records. (**C**) Modeled Southern Hemisphere ice volume (*30*). Purple line is the LOWESS smoothing with a 100 kyr window. (**D**) Black dots indicating the boundary of marine isotope stages (MIS) (*1*). (**E**) Time series of hydrogenous ε_Nd_ (filled black circles) from Site U1541 (this study) and benthic δ^13^C (green line) from ODP Site 1123 (*5*). (**F**) Atmospheric pCO_2_ records from (*9*, *44*–*47*). The gray bars and marine isotope stages represent the interglacial and glacial respectively. (**G**) IRD flux records from the Scotia Sea (binned at 5 kyr intervals) showing prominent increase in IRD starting at 1.8 Ma (*28*) (transition period shown by gray bar).

To gain more insight in the pre-MPT Southern Ocean, we compare our ε_Nd_ records from Site U1541 with Mg/Ca-derived Bottom Water Temperature (BWT) reconstructions of ODP Sites 607 (*26*, *27*) in the North Atlantic and 1123 (41.8°S, 171.5°W, 3290 m water depth) in the SW Pacific (*5*). Both Sites 607 and 1123 have similar latitudinal position in their respective hemispheres, and we assume that their BWT evolution approximates the polar conditions in each hemisphere. The Southern Hemisphere BWT record is consistently cooler during the pre-MPT in comparison to the North Hemisphere (Fig. 4B). Site 1123 BWT sharply cooled at 1.425 Ma and continued to remain colder than late Pleistocene average BWTs for another 200 kyr. This period coincides with the gradual increase in ε_Nd_ at Site U1541, where every subsequent glacial period became more radiogenic until MIS 38, suggesting diminishing contribution of NCW (Fig. 4E). Together, these proxies suggest that high latitude Southern Hemisphere climate and deep ocean circulation were linked leading up to the MPT.

Evidence in favor of Antarctic ice buildup in the pre-MPT can be found in Southern Ocean Ice Rafted Debris (IRD) records (*28*). IRD records from the Atlantic section of the Southern Ocean (Scotia Sea) (*28*) indicate that by ~1.8 Ma, the AIS expanded and transitioned from terrestrial to become glaciomarine. In context of Antarctica, glaciomarine ice sheets are those that have reached the ocean. Once ice sheets reach the oceans, they are heavily influenced by sea level fluctuations and begin to calve, forming icebergs that eventually melt to release the entrapped IRD. This process can, in turn, translate Southern Hemisphere climate variations to the Northern Hemisphere through the influence of Southern Ocean iceberg melt on AMOC. An IRD study in the South Atlantic argued that glacial conditions allow for icebergs from the Weddell Sea to travel further north from Antarctica into subtropical regions before melting, a process termed “southern escape” (*29*). As icebergs melt, the lens of freshwater reduces the density in the upper AMOC cell, inhibiting surface return flow to the North Atlantic and suppressing NADW formation. With time, reduced NADW production would lead to a more radiogenic Southern Ocean ε_Nd_ signature, as is supported by our data across MIS 46-38 (Fig. 2). Starr *et al.* (*29*) also reported model results that showed twice as much meltwater in the South Atlantic between 0° and 50°E during the Last Glacial Maximum when compared to preindustrial conditions. This shows that this mechanism can sufficiently change the buoyancy budget of the Southern Ocean. A recent reconstruction indicates an increasing trend of Southern Hemisphere ice volume as early as 2 Ma (*30*) (Fig. 4C). Therefore, while there is prior evidence of marine-based AIS (*31*), we hypothesize that by 1.8 Ma, AIS attained a critical size threshold to become marine-terminating across subsequent glacial cycles. After this time, glacial periods experienced iceberg release and southern escape, with melting ultimately introducing fresh water into the AMOC return flow in the South Atlantic. This mechanism started at ~1.4 Ma, weakening AMOC with every glacial cycle until AMOC reached its weakest state at MIS 38 as indicated by the elevated ε_Nd_ at Site U1541 (Fig. 2).

### Role of the Southern Hemisphere in the MPT

With our data and other proxy evidence from both the Southern and Northern Hemispheres, a sequence of events leading to the MPT can be proposed. First, long-term Pleistocene cooling and resulting Antarctic ice growth to marine margins led to ice calving and subsequent melting in the Southern Hemisphere, initiating a long-term AMOC slowdown. Modern observational records and modeling outputs indicate that an AMOC slowdown or collapse would result in Northern Hemisphere cooling [e.g., (*32*) and references therein]. Northern Hemisphere cooling is evident after 1.25 Ma in stacked records of sea surface temperature (*30*). In addition to cooling, the supply of moisture is a necessary prerequisite for ice growth. Modeling studies support the idea that a cooler Southern Hemisphere can push the intertropical convergence zone northward, resulting in concomitant changes in Hadley cell dynamics and net moisture transport to the Northern Hemisphere (*33*, *34*). Once both moisture and temperature conditions became favorable, the Northern Hemisphere ice sheets entered a mode of net ice gain.

If this is true, how do we reconcile the optimal conditions for Northern Hemisphere ice growth at ~1.25 Ma with no evidence of for decrease in atmospheric CO_2_ (Fig. 4G) or concomitant increase in deep ocean carbon storage until 0.9 Ma? (*7*) We suggest, starting at ~1.4 Ma, surface freshening due to iceberg melting near Antarctica and subsequent slowdown of AMOC during glacial periods would support shallow (*35*) and deep Southern Ocean stratification, preconditions necessary to store carbon in the deep Southern Ocean (*11*, *29*). The benthic δ^13^C record at Site 1123 shows evidence of gradual decline (Fig. 4E) with every consecutive glacial that coincides with Site U1541 ε_Nd_ record. The decreasing δ^13^C could indicate AMOC slowdown when the glacial pacing was still 41 kyr as observed in the benthic δ^18^O record (*1*). The quasi-symmetrical glacials during the 41 kyr world were primarily driven by astronomical forcing (*36*) that allowed equivalent time for ice growth and decay. During this pre-MPT period, we suggest that the accumulation of carbon in the deep ocean due to AMOC slowdown was short-lived. This could explain why, in spite of seemingly having the necessary mechanisms to store carbon in the deep Southern Ocean, lower glacial atmospheric CO_2_ in proxy records is not observed during the 41 kyr world (Fig. 4H).

In contrast, the late Pleistocene glacial periods lasted for ~100 kyr and were characterized by asymmetrical features of longer ice growth (~90 kyr) and fast deglaciation (~10 kyr). This prolonging of glacial period in the later Pleistocene holds the key, as stratification in the deep ocean would have been sustained for a longer period. Although surface ocean carbon uptake and export are relatively rapid processes (*9*), rather, the prolonging of deep stratification is needed to maximize the regenerated nutrient content of the deep ocean and thus maximize its carbon sequestration potential. On the basis of our data and other subsidiary proxy evidence from the Southern Hemisphere, we posit that 1.25 Ma is the turning point (Supplementary Materials) when environmental factors favored synchronization of Northern and Southern Hemisphere climate conditions for ice volume growth. By ~0.9 Ma, ice volume at both poles reached a critical size such that the glacial cycles started to become asymmetrical but did not reach the late Pleistocene configuration for another 250 kyr when the first 100 kyr cycle arrived. At ~0.9 Ma, dissolved oxygen in glacial deep Atlantic started diminishing due to extensive ice cover in the deep-water formation region in the Labrador Sea (*37*), suggesting reduced gas exchange with the deep ocean, a condition that can favor deep ocean carbon storage. This shift in symmetry of glacial cycles, with an extended bipolar glaciation phase, helped to lengthen the residence time of carbon in the deep ocean, leading to the lower glacial CO_2_ levels (*9*) as suggested by some previous studies.

## MATERIALS AND METHODS

### Location, hydrography, and site selection rationale

The study area is in the central South Pacific where sediment cores were collected during the International Ocean Discovery Program (IODP) Expedition 383 (Fig. 1A). IODP Site U1541 (54°13′S, 125°25′W, water depth 3604 m) is located ~300 km from the western flank of the EPR and ~185 km north of the sub-Antarctic front (SAF), the outermost front of the Antarctic circumpolar current. While multiple holes were drilled at Site U1541 to retrieve a continuous record, shipboard data (*38*) show that a gap of ~1 m (~40 kyr) is present at about 1.1 Ma. Modern hydrography suggests that the modern location of Site U1541 is bathed by LCDW (Fig. 1B). The LCDW in the South Pacific represents a remnant of saline NADW (albeit modified) and North PDW (NPDW), a low-oxygen water mass that often occupies depths between 1500 and 3500 m (*39*). Therefore, Site U1541 is well positioned to capture deep ocean circulation changes before, during, and after the MPT expressed as relative proportions of Atlantic and Pacific sourced water masses in the South Pacific basin.

### Age model

The shipboard age model constructed on the basis of biostratigraphic and paleomagnetic age-control points (*38*) was later improved by Middleton *et al.* (*39*) using high-resolution benthic δ^18^O generated using a combination of genera *Cibicidoides*, *Cibicide*s, and *Uvigerina*. The high-resolution benthic δ^18^O (*39*) was aligned with the probabilistic stack using the Hidden Markov Model algorithm of Lin *et al.* (*40*) and Ahn *et al.* (*41*). Reported age model uncertainties emanate from both automated probabilistic alignment (1 to 3 kyr) and choice of tuning target (2 to 6 kyr) (*39*). In this study, we use the published age model of Middleton *et al.* (*39*), with an average sedimentation rate of ~3 cm/kyr over our study interval.

### Nd isotope analysis

Fossilized bio-phosphate (fossil fish teeth/debris) and Fe-Mn oxy-hydroxide encrusted planktonic foraminifera used for Nd isotope analyses were hand-picked (from the >63 μm fraction) under a microscope, followed by careful physical cleaning of detrital particles, which is critical for obtaining the pure paleo seawater signal. Physical cleaning typically comprised multiple rinses and sonication in Optima grade methanol, followed by multiple rinses in deionized water. The majority of samples used to generate the continuous record of ε_Nd_ were fish teeth; however, foraminifera were used whenever samples lacked bio-phosphate materials. The foraminifera were crushed with glass slides to break open the inner chamber before proceeding with the physical cleaning. The cleaned broken foraminifera fragments were examined under a microscope to ensure that no clay fragments were left in the sample.

Following the physical cleaning, the samples were subjected to dissolution using 1:1 Optima grade concentrated Nitric and Hydrochloric acid for fossil fish teeth, and 5% Optima grade acetic acid for foraminifera samples. Rare earth element separation was done using 100-μl shrink-fit Teflon columns, loaded with Eichrom TRUspec resin (100- to 150-μm mesh). Further isolation of Nd was done using calibrated Teflon columns preloaded with Eichrom LNspec resin (50- to 100-μm mesh). Samples were analyzed for Nd isotopes at Pennsylvania State University (PSU), using a Thermo Fisher Scientific Neptune Plus multicollector–inductively coupled plasma mass spectrometer (MC-ICPMS). A desolvating sample introduction system (Apex nebulizer) was used for sample introduction into the MC-ICPMS. Measured ^143^Nd/^144^Nd ratios are corrected using ^146^Nd/^144^Nd = 0.7219 and an exponential mass fractionation law. Mean ^143^Nd/^144^Nd of measured JNdi-1 for a given analytical session and the published ^143^Nd/^144^Nd of JNdi-1 [=0.512115; (*42*)] were used to report normalized ^143^Nd/^144^Nd ratios of the samples. All samples were bracketed by JNdi-1 that were concentration matched, that is, the JNdi-1 concentration was adjusted to the sample concentration. Repeat analyses of 20-ng international standard JNdi-1 by our group at PSU yielded reproducibility within ±0.30 ε_Nd_ units (2SD, *n* = 19). A small subset of the reported samples were analyzed at the Lamont-Doherty Earth Observatory (LDEO), Columbia University. LDEO and PSU have identical instrumental and sample introduction setup. Cross calibration between labs using repeat analysis of same samples showed agreement within the external reproducibility (2SD) of JNdi-1.

## Supplementary Materials

### This PDF file includes:

Supplementary Text Figs. S1 to S5 Table S1 References
