# Supplementary material for: Southern Hemisphere initiation of the mid-Pleistocene transition
Source: Sci Adv. 2026 Mar 4;12(10):eaea6811. doi: 10.1126/sciadv.aea6811 (PMC12959411; doi:10.1126/sciadv.aea6811)
Supplement: Supplementary file 1 — Supplementary Text Figs. S1 to S5 Table S1 References [file sciadv.aea6811_sm.pdf]

Science Advances
AAAS

Supplementary Materials for

Southern Hemisphere initiation of the mid-Pleistocene transition

Chandranath Basak *et al.*

Corresponding author: Chandranath Basak, cbasak@udel.edu

Sci. Adv. **12**, eaea6811 (2026)
DOI: 10.1126/sciadv.aea6811

This PDF file includes:

Supplementary Text
Figs. S1 to S5
Table S1
References

Supplementary Text

Proxy description and existing interpretive framework

Neodymium (Nd) isotopes can be used to trace present and past ocean circulations, because its variability in the earth mainly reflects the radioactive decay of ^{147}Sm to ^{143}Nd ($t_{1/2}$ =106 Ga) over geological time, rather than temperature, redox, air-sea exchange, or biology. Sm/Nd ratios show a small variation in continental rocks and are low compared to the mantle. As a result, the continents have lower ^{143}Nd/^{144}Nd values than the mantle, and there is a systematic relationship between Nd isotope ratios and crustal age, with older continent materials showing lower values. Nd isotopes are often presented as ε_{Nd}, defined as the deviation of the ^{143}Nd/^{144}Nd ratio in a sample from the average value of a chondritic uniform reservoir (=0.512638, (*48*)) in parts per 10^4. The low and high ε_{Nd} values represent unradiogenic and radiogenic Nd isotope ratios respectively.

Early measurements of ε_{Nd} in the ocean showed systematic variations in seawater in different ocean basins that were recorded in hydrogenetic (i.e., derived from seawater) ferromanganese crust deposits. Subsequently, Nd isotopes in the global ocean has been largely defined as a two end-member system (i.e., the North Atlantic and North Pacific (*49*)) reflecting the geology of surrounding continents (*18*, *50*). The old Archean continental rocks in Canada and Greenland with extremely low ε_{Nd} values make the North Atlantic deep waters highly unradiogenic, while young volcanic rocks around the Pacific with much higher εNd values makes the deep Pacific waters radiogenic. Ferromanganese crust data also indicated that the end-members remained relatively constant at least for the last 2 Myr (*6*). Intermediate locations (for example in the Southern Ocean) are then explained as linear mixing between these two end-members. Measurements of dissolved Nd isotopes in modern seawater support this general interpretive framework where Nd isotope ratios are lowest in the North Atlantic ($\varepsilon_{Nd} = -10$ to -14 (*12*, *13*)), highest in the North Pacific (ε_{Nd} = 0 to -4 (*14*)) and intermediate in the Southern Ocean ($\varepsilon_{Nd} = -8$ to -9 (*16*, *51*, *52*)). In this study, Nd isotopes will be used as a tracer of water mass mixing.

Proxy complications

Over the years, the conventional interpretive framework which suggests Nd isotopes in the global ocean behave conservatively has been challenged and various oceanic sources of Nd have been introduced. Nd added through partial dissolution of aeolian dust in the surface ocean, ridge exchange and hydrothermal input of Nd near mid-oceanic ridges, near shelf boundary exchange as a source of Nd, and more recently pervasive benthic flux of Nd are noteworthy. Dust input is particularly important as dust borne Fe is thought to be one of the primary sources of micronutrient that controls glacial-interglacial surface productivity in the Southern Ocean (*9*). While dust is important for surface productivity, Carter *et al.*(*53*) used simple mass balance calculations to rule out the role of dust in perturbing the deep-water Nd inventory in the South Pacific to influence the seawater Nd isotopes. Therefore, it is reasonable to exclude dust as a viable source that can explain Nd isotope changes in the deep South Pacific. Another possible source of open ocean Nd could be the influence of mid-ocean ridge materials which are often made up of highly reactive rocks carrying a radiogenic Nd isotopic signature. High Nd isotope ratios added through exchange between seawater Rare Earth Elements and ridge materials can propagate into the shallow and deep waters, a mechanism recognized as ridge exchange (e.g., (*54*)). Evidence in support of this mechanism is relatively rare, with one reported occurrence at a station proximal to the East Pacific Rise (*54*). Similarly, trace metal enriched hydrothermal fluids can be considered a potential source of Nd. A recent study by Basak *et al.*(*55*) showed no observable exchange between the East Pacific Rise (EPR) and samples collected along the GEOTRACES Pacific section (GP16) that extensively crossed EPR and sampled hydrothermal plumes off EPR (*56*). This is consistent with previous

studies which showed that hydrothermal input is not a significant source of Nd to the global ocean (*57–59*). Dissolution/exchange with sediments along continental margins (cf. boundary exchange) is particularly discussed as a substantial source of Nd to the global ocean (*60*, *61*). In this regard it is noteworthy to mention that western Pacific margins are recognized sources of non-conservative Nd to the seawater, yet the sources are not sufficient to completely erase the effect of water mass mixing and deep circulation on downcore ε_{Nd}. This margin effect is also reported to fade away from the shelf (*60*). Therefore, a shelf proximal site will still have the signature of changes in deep ocean circulation, however, muted. It will be an oversimplification to assume that open ocean Sites are completely devoid of local influence, however it will be reasonable to assume that influence of non-conservative Nd is not dominant and hence the signal that is due to water mass mixing is more prominent. In this regard, Site U1541 is ideal as it is situated in the open ocean and far away from the continents, so boundary exchange which is predominantly a margin process can be largely excluded. Similarly, we do not expect lithogenic materials brought in via Antarctic glacial outflow to affect our hydrogenous Nd isotope records.

For over two decades, ε_{Nd} values preserved in ferromanganese (Fe-Mn) oxide precipitates on foraminifera and fossilized bio-phosphate (fossil fish teeth/debris) in sediment cores have been used to reconstruct past changes in meridional overturning circulation (e.g., (*11*, *43*, *62*, *63*), and many others). The rationale behind using these archives as a paleocirculation proxy is based on the premise that the preserved εNd signal represents deep ocean water at the core sites (*64*). Recently, based on a handful of observational data a new interpretation framework has been proposed which claims that benthic Nd flux or preferential dissolution of reactive phases in the sediment are primary controls on setting the bottom water and hence the authigenic ε_{Nd} value (*65–67*). While this is a new and evolving interpretive framework, supporting data such as identification and quantification of such a benthic flux is largely absent for the modern ocean, let alone in the paleo record. Therefore, it is not clear how such a flux would impact the Nd isotopic composition of bottom waters. We consistently observe that large basin-scale deep ocean circulation through time is well represented by down core hydrogenous (i.e., seawater derived) Nd isotope data, in both the Atlantic and Pacific ((*43*, *63*, *68*), and many others). For the purpose of this study, our primary approach would be to interpret this proxy in the light of conservative mixing between water masses. As benthic flux quantification improves, the interpretation can be revisited.

Interlaboratory comparison

All foraminifera and fossil teeth samples analyzed for $^{143}Nd/^{144}Nd$ isotopes were processed at the University of Delaware. Several (n=11) procedural blanks were monitored for Nd during the course of the study and the maximum measured blank value of 28 pg of Nd, hence negligible, and therefore no blank correction was applied. The reported $^{143}Nd/^{144}Nd$ data were analyzed, in part, at Pennsylvania State University (PSU) and the Lamont-Doherty Earth Observatory (LDEO) at Columbia University. Although both labs used a Thermo Scientific Neptune Plus multicollector inductively coupled plasma mass spectrometer (MC-ICPMS) with similar methods and instrumental setups, interlaboratory comparison is essential to check for potential offsets and ensure data comparability. To this end, five random samples (with sufficient material for duplicate analysis) were analyzed at both PSU and LDEO (fig. S1). The interlaboratory comparison shows good agreement (R^2= 0.9) between the labs, indicating that the data generated at both institutions can be interpreted without the need for correction.

Comparing ε_{Nd} time-series across global oceans

Comparing the ε_{Nd} time-series along the Atlantic Meridional Overturning Circulation (AMOC) flowpath is an important step to monitor changes that happened in the deep ocean circulation during the MPT and the time leading upto the MPT (fig. S2 and S3). Other than ODP 929, which is located at the boundary of NADW and AABW, all other Atlantic records are located within NADW (fig. S2). While resolutions of the records are variable making one-to-one comparison difficult, the large-scale observations can still be made with confidence.

1. The low interglacial and high glacial ε_{Nd} values are observed in all records. This would suggest a systematic variation a) the volume of NADW production and propagation along AMOC pathway, or b) changes in North Atlantic end-member driven by northern hemisphere ice sheet weathering, or a combination of (a) and (b).
2. MIS 38, and MIS 22 are prominent excursions that can be tracked in all records.
3. There is an increase in glacial-interglacial hydrogenous ε_{Nd} values starting at 1.4 Ma that continued until MIS 38.
4. The step change in median ε_{Nd} values before and after MIS 22 as identified in the North Atlantic(*43*) is not observed in the South Atlantic (*11*) or South Pacific (this study).

To summarize, the major features discussed in the paper are global signatures and not local to the Site U1541.

End-member mixing model

The choice of end-members in binary mixing model calculations for Nd isotopes has been a contentious topic as long as the proxy has been used. In this study, we follow a modified version of the approach implemented by Yehudai et al. (*69*). The binary mixing model assumes two end-member water masses: Pacific Deep Water (PDW) and North Atlantic Deep Water (NADW). Any linear mixing model that involves radiogenic isotopes require three parameters viz. isotope and concentrations of end-members, and proportion of end-members. Based on observational records and a literature survey, we assigned a range of Nd isotope and concentration values for both end-members. Specifically, the Nd isotope for NADW (the northern end-member) ranges between -10.5 and -14.5 (*21*), and the corresponding Nd concentration ranges between 17 and 23 µmol/kg (*70*). For PDW (the Pacific end-member), the prescribed Nd isotope range is between -3 and -4 (*14*), and the Nd concentration ranges between 15 and 47 µmol/kg. Given the assigned values, the model has an implicit assumption that the N. Pacific end-member ε_{Nd} did not change much. This is not an unreasonable assumption as PDW is a recycled water where ε_{Nd} is attained during its transit form the south via interaction with volcanic rocks, and continental margins. Since PDW does not form by sinking of surface water, surface processes such as ice-sheet related weathering input is not expected to directly alter the ε_{Nd} of PDW.

We allowed the model to randomly assign the proportions of Atlantic and Pacific water masses to be such that they add up to 1. The mixing model followed equation 1.

$$\varepsilon_{Nd\,(calculated)} = \frac{\varepsilon_{Nd\,(NCW)} * [Nd]_{(NCW)} * P_{(NCW)} + \varepsilon_{Nd\,(PDW)} * [Nd]_{(PDW)} * (1-P)_{(PDW)}}{[Nd]_{(NCW)} * P_{(NCW)} + [Nd]_{(PDW)} * (1-P)_{(PDW)}} \quad \text{Eq. 1}$$

The NCW and PDW are the northern component water and Pacific deep water respectively and P indicates the proportion of each water mass. The model was set to randomly select each parameter (i.e., ε_{Nd}, [Nd], P) from the assigned range of end-members to calculate Nd isotope (i.e., $\varepsilon_{Nd\,(calculated)}$). The model was set up to run 1000 iterations, with each iteration randomly selecting Nd isotopes, concentrations, and mixing proportions from the prescribed ranges. This process was repeated for each observational data point reported for Site U1541 in this study. Thus, each time

step resulted in 1000 calculated Nd isotope values. A modeled value was accepted if it agreed within ±0.3 epsilon envelope of the measured values at that time step. As a result, only a subset of the 1000 runs were valid. For each parameter space, the end-member values of the 'valid' subset are considered as 'acceptable'. There is no unique value of a given parameter for each time step. We took the mode of the valid parameter values at each time step as the representation of the model parameters that best reflect the end-member water masses and their mixing proportions. Before we interpret the model results (fig. S4), it is important to point out that the implemented model and the results are only used to obtain a broad scale estimate of end-member change and should not be considered to represent the exact evolutionary history of the end-members.

Importance of 1.25 Ma and northern hemisphere ice growth

Tzedakis *et al.* (*71*) utilized integrated Northern Hemisphere summer insolation and a time-dependent discount metric to show that interglacials occurred at every obliquity peak prior to 1.25 Ma. This scenario suggests that the Northern Hemisphere ice sheets were relatively small and responded linearly to insolation forcing. Right after 1.25 Ma (and became more prominent after 1 Ma), deglaciation began to occur only after two or more insolation peaks, indicating that the ice sheets had grown to medium size, were less sensitive to insolation forcing, and were capable of surviving consecutive summer insolation peaks—resulting in net growth (fig. S5). A similar argument involving 'small' and 'medium' ice sheet thresholds was proposed in a modeling framework by Berends et al. (*72*) and refs. therein. More recently, An *et al.*(*30*) compiled multi-proxy records from sites across the Northern Hemisphere, all showing evidence of rapid ice sheet growth after ~1.25 Ma. Our Nd isotope data and model-based North Atlantic end-member values also suggest a turning point around 1.25 Ma (Fig. 3), when ice sheet–driven changes in deep ocean circulation began.

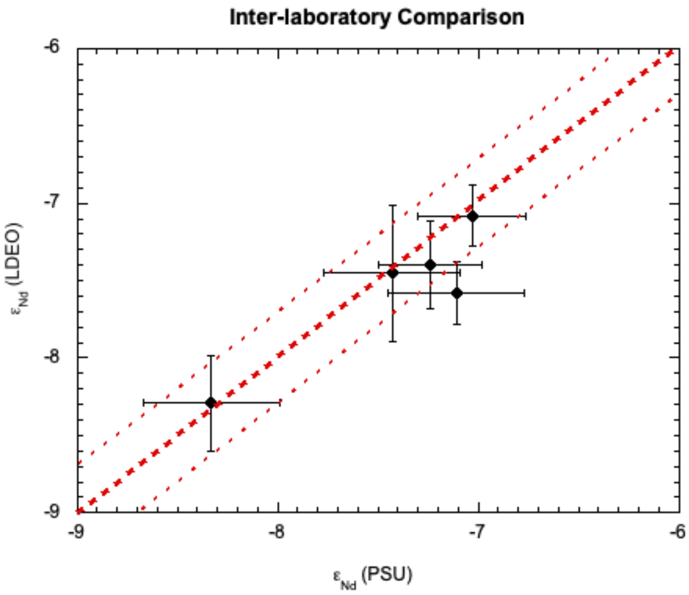

Fig. S1.

A plot showing the inter-laboratory comparison of reported ε_{Nd} values analyzed at PSU and LDEO. The bold red dashed line represents the 1:1 line, and the surrounding dashed red lines represent the typical long-term analytical error of 0.3 ε unit. The reported error bars represent the maximum error. Error bars larger than 0.3 ε units indicate that the internal errors exceed the long-term external error (2SD) for that particular sample.

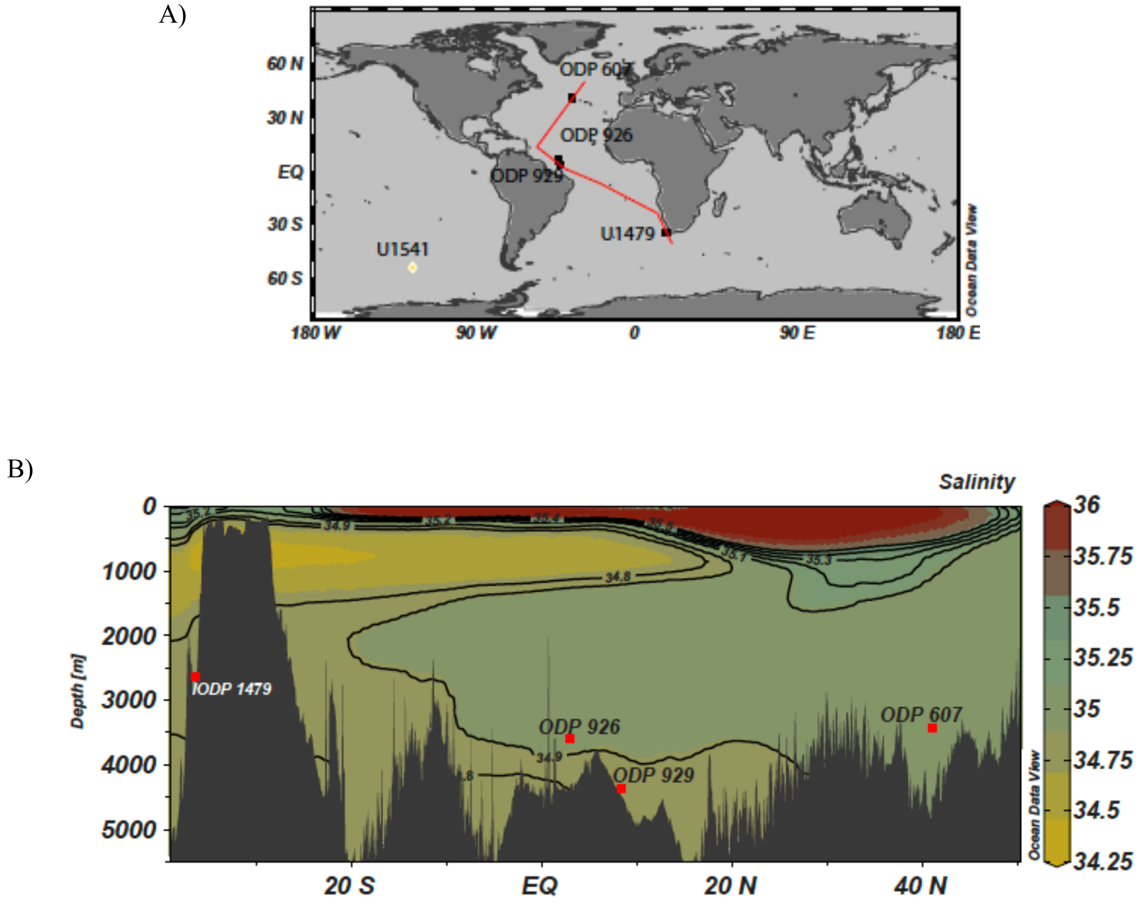

Fig. S2.

Figure showing Site locations and water masses of ε_{Nd} time-series encompassing 1.5-0.5 Ma. A) Map of Site locations in the North (OPD 607,(*43*)), Equatorial (ODP 929 (*73*), ODP 926 (*8*, *17*)), South Atlantic (U1479 (*11*)), and South Pacific oceans (U1541, this study). Red line indicates the profile shown in (B). B) Salinity vs. depth along the Atlantic Ocean. Except ODP 929, all sites situated within the core North Atlantic Deep Water (NADW). ODP 929 is located along the boundary of NADW and Antarctic Bottom Water.

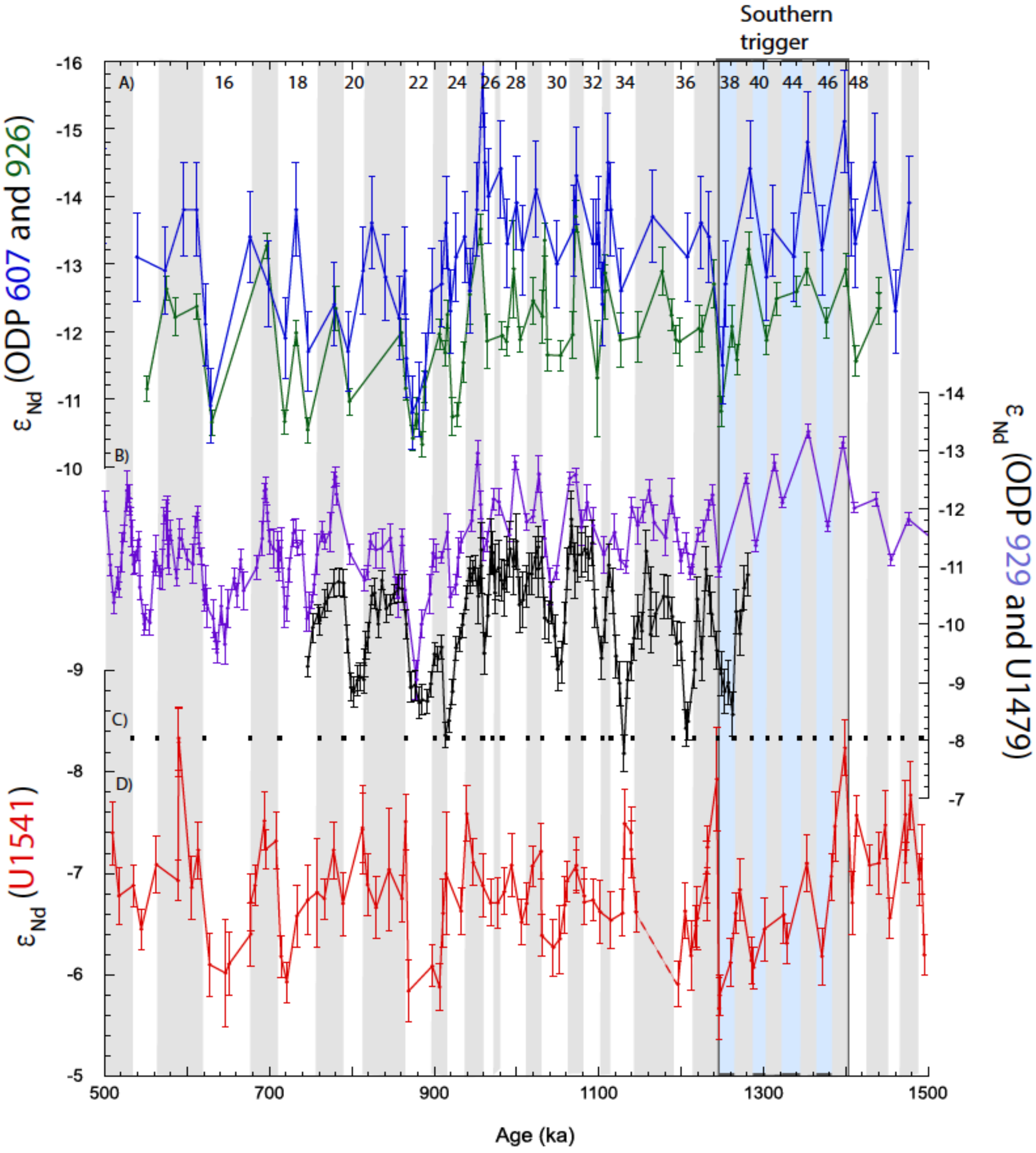

Fig. S3.

Plot showing hydrogenous ε_{Nd} time-series along the Atlantic Ocean in comparison to central South Pacific. A, B) The Atlantic ε_{Nd} time-series (*8*, *11*, *17*, *43*, *73*). C) Black dots indicating the boundary of Marine Isotope Stages (MIS) (*1*). D) Down core ε_{Nd} time-series at Site U1541 in the central South Pacific (this study). The blue shaded region between MIS 46-38 indicates timing of possible Southern Hemisphere trigger. All records are in their respective age models which are all tuned to LR04 benthic stack (*1*).

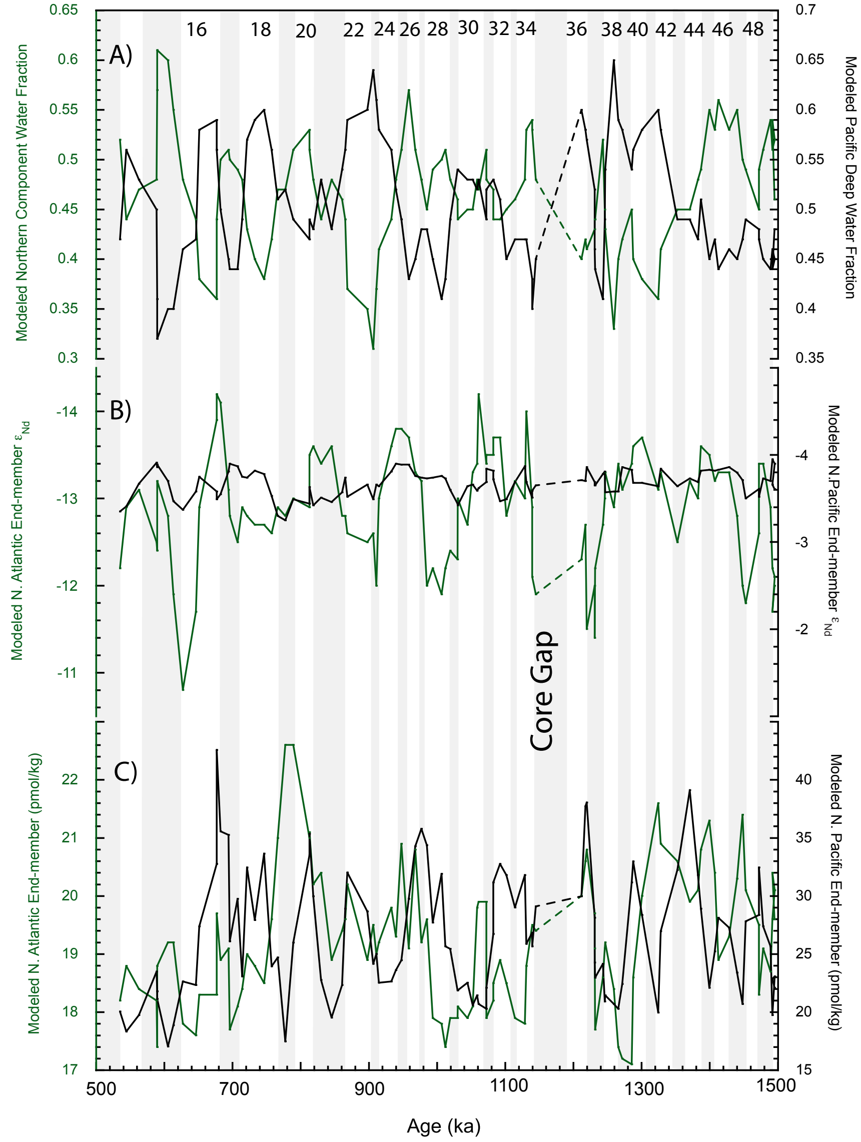

Fig. S4.
Plots show three point running mean of the representative parameter values from the mixing model used in this study. Modeled North Atlantic (in black) and Pacific (in green) A) water mass fraction, B) end-member ε_{Nd}, and C) Nd concentrations. Core gap is shown by broken lines. Grey bars are interglacials. Numerals are glacials.

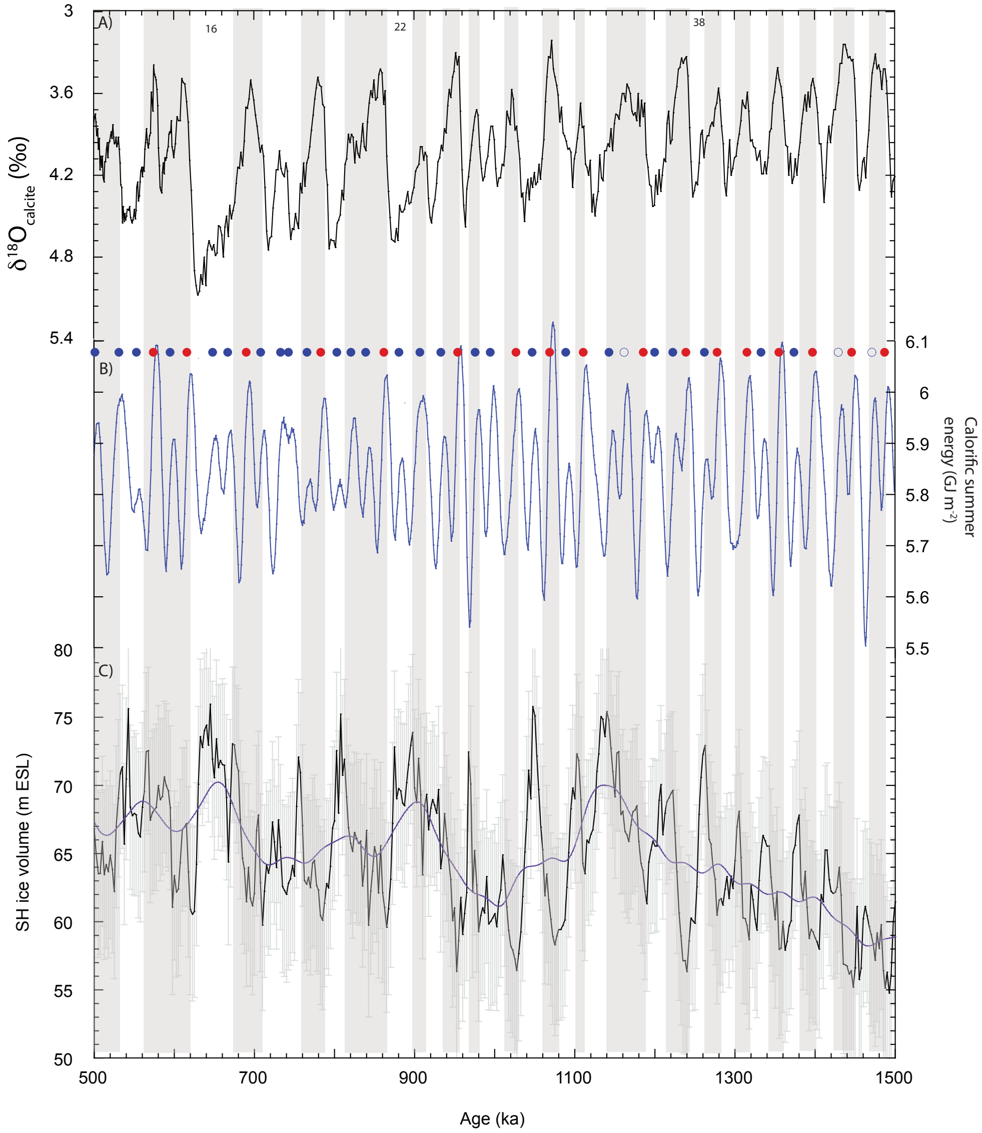

Fig. S5.

Proxy and model climate records showing relative timing of ice growth in two hemispheres. A) benthic $\delta^{18}O$ between LRO4 global stack (*1*). B) Solar insolation peak calculated from caloric summer energy (*71*). The filled red circles are insolation peaks that can produce interglacials and filled blue circles indicate insolation peaks that cannot give rise to interglacials. Empty blue circles are continued-interglacials. C) Modeled Southern Hemisphere ice volume (*30*). Purple line is the LOWESS smoothing with a 100 kyr window.

Table S1
Neodymium isotope data from Site U1541.

Sample Name	Depth CCSF (m)[1]	Age (ka)	Normalized $^{143}Nd/^{144}Nd$	ε	Internal error (2SE)	External error (2σ)	Highest error[2]	Archive[3]	Lab[4]
383-U1541B-2H,3W,132-134	14.1	509.2	0.512259	-7.40	0.10	0.31	0.31	FD	PSU
383-U1541B-2H,4W,4-5	14.3	517.1	0.512290	-6.78	0.14	0.28	0.28	FD	PSU
383-U1541B-2H,4W,37-39	14.6	534.9	0.512285	-6.88	0.18	0.21	0.21	FD	PSU
383-U1541B-2H,4W,59-60	14.9	543.9	0.512307	-6.45	0.12	0.20	0.20	FD	PSU
383-U1541B-2H,4W,114-115	15.4	562.5	0.512274	-7.09	0.16	0.28	0.28	FD	PSU
383-U1541B-2H,5W,42-43	16.1	588.7	0.512283	-6.93	0.06	0.20	0.20	FD	PSU
383-U1541B-2H,5W,45-47*	16.1	589.6	0.512213	-8.29	0.15	0.34	0.34	FD	LDEO
383-U1541B-2H,5W,45-47	16.1	589.6	0.512211	-8.33	0.12	0.31	0.31	Foraminifera	PSU
383-U1541B-2H,5W,95-97	16.6	605.2	0.512286	-6.86	0.09	0.31	0.31	FD	PSU
383-U1541B-2H,5W,117-118	16.9	613.4	0.512267	-7.23	0.13	0.28	0.28	FD	PSU
383-U1541B-2H,5W,145-147	17.1	626.9	0.512325	-6.10	0.18	0.31	0.31	FD	PSU
383-U1541B-2H,6W,38-39	17.6	646.1	0.512329	-6.02	0.12	0.53	0.53	FD	PSU
383-U1541B-2H,6W,46-48	17.6	651.0	0.512325	-6.11	0.09	0.31	0.31	FD	PSU
383-U1541B-2H,6W,96-98	18.1	676.7	0.512310	-6.40	0.17	0.31	0.31	Foraminifera	PSU
383-U1541B-2H,6W,96-98*	18.1	676.7	0.512293	-6.72	0.15	0.28	0.28	FD	PSU
383-U1541B-2H,6W,108-109	18.3	682.3	0.512285	-6.88	0.11	0.20	0.20	FD	PSU
383-U1541C-3H,3W,14-16	18.6	694.0	0.512253	-7.52	0.14	0.28	0.28	FD	PSU
383-U1541C-3H,3W,21-22	18.7	695.9	0.512267	-7.24	0.13	0.20	0.20	FD	PSU
383-U1541C-3H,3W,64-66	19.1	707.4	0.512263	-7.32	0.17	0.28	0.28	Foraminifera	PSU
383-U1541C-3H,3W,86-87	19.4	714.3	0.512321	-6.18	0.12	0.20	0.20	FD	PSU
383-U1541C-3H,3W,114-116	19.6	721.0	0.512334	-5.93	0.12	0.20	0.20	FD	PSU
383-U1541C-3H,4W,14-16	20.1	732.8	0.512301	-6.58	0.15	0.31	0.31	FD	PSU
383-U1541C-3H,4W,64-66	20.6	746.2	0.512292	-6.74	0.15	0.34	0.34	Foraminifera	LDEO
383-U1541C-3H,4W,114-116	21.1	757.5	0.512289	-6.81	0.15	0.53	0.53	Foraminifera	PSU
383-U1541C-3H,5W,14-16	21.6	766.2	0.512292	-6.75	0.11	0.20	0.20	FD	PSU
383-U1541C-3H,5W,64-66	22.1	777.3	0.512267	-7.23	0.13	0.28	0.28	Foraminifera	PSU
383-U1541B-3H,2W,59-61	22.6	788.9	0.512294	-6.70	0.14	0.31	0.31	FD	PSU

383-U1541B-3H,3W,2-3	23.3	812.6	0.512256	-7.45	0.13	0.34	0.34	Foraminifera	PSU
383-U1541B-3H,3W,2-3*	23.3	812.6	0.512257	-7.43	0.20	0.44	0.44	FD	PSU
383-U1541B-3H,3W,12-13	23.4	818.3	0.512285	-6.89	0.31	0.27	0.31	FD	LDEO
383-U1541B-3H,3W,40-42	23.6	829.7	0.512296	-6.66	0.14	0.31	0.31	Foraminifera	PSU
383-U1541B-3H,3W,90-92	24.1	845.0	0.512277	-7.04	0.18	0.60	0.60	FD	PSU
383-U1541B-3H,4W,18-19	24.5	860.7	0.512292	-6.75	0.16	0.23	0.23	FD	PSU
383-U1541B-3H,4W,28-29	24.6	865.5	0.512253	-7.51	0.25	0.27	0.27	FD	LDEO
383-U1541B-3H,4W,36-38	24.6	868.4	0.512338	-5.84	0.15	0.31	0.31	FD	PSU
383-U1541B-3H,4W,136-138	25.6	897.5	0.512326	-6.09	0.16	0.21	0.21	FD	PSU
383-U1541B-3H,5W,15-16	25.9	906.4	0.512337	-5.88	0.17	0.23	0.23	FD	PSU
383-U1541B-3H,5W,25-26	26.0	910.8	0.512299	-6.61	0.18	0.34	0.34	Foraminifera	LDEO
383-U1541B-3H,5W,38-40	26.2	914.6	0.512279	-7.00	0.25	0.60	0.60	FD	PSU
383-U1541B-3H,5W,75-76	26.5	933.0	0.512298	-6.63	0.16	0.23	0.23	FD	PSU
383-U1541B-3H,5W,95-96	26.7	939.5	0.512249	-7.59	0.17	0.27	0.27	Foraminifera	LDEO
383-U1541B-3H,5W,115-116	26.9	947.5	0.512274	-7.11	0.18	0.23	0.23	FD	PSU
383-U1541B-3H,5W,138-140	27.2	958.6	0.512285	-6.88	0.09	0.31	0.31	FD	PSU
383-U1541B-3H,6W,18-19	27.4	968.1	0.512294	-6.71	0.13	0.23	0.23	FD	PSU
383-U1541B-3H,6W,41-43	27.6	976.9	0.512294	-6.71	0.21	0.25	0.25	Foraminifera	PSU
383-U1541B-3H,6W,68-69	27.9	985.0	0.512289	-6.82	0.14	0.23	0.23	FD	PSU
383-U1541B-3H,6W,91-93	28.1	993.7	0.512275	-7.08	0.11	0.31	0.31	FD	PSU
383-U1541C-4H,4W,113-114	28.4	1006.4	0.512304	-6.52	0.10	0.23	0.23	FD	PSU
383-U1541C-4H,4W,134-135	28.6	1012.1	0.512295	-6.70	0.12	0.20	0.20	FD	PSU
383-U1541C-4H,5W,8-9	28.9	1019.3	0.512275	-7.07	0.13	0.20	0.20	FD	PSU
383-U1541C-4H,5W,36-38	29.1	1029.8	0.512268	-7.22	0.17	0.28	0.28	Foraminifera	PSU
383-U1541C-4H,5W,38-39	29.2	1030.2	0.512311	-6.39	0.14	0.20	0.20	FD	PSU
383-U1541C-4H,5W,86-88	29.6	1044.5	0.512317	-6.27	0.13	0.28	0.28	Foraminifera	PSU
383-U1541C-4H,5W,113-114	29.9	1052.1	0.512312	-6.35	0.19	0.34	0.34	FD	PSU
383-U1541C-4H,5W,136-138	30.1	1058.7	0.512295	-6.69	0.20	0.28	0.28	Foraminifera	PSU

383-U1541C-4H,5W,143-144	30.2	1060.7	0.512283	-6.92	0.11	0.20	0.20	FD	PSU
383-U1541C-4H,6W,23-24	30.5	1072.2	0.512275	-7.08	0.19	0.27	0.27	FD	LDEO
383-U1541C-4H,6W,23-24	30.5	1072.2	0.512278	-7.03	0.12	0.20	0.20	Foraminifera	PSU
383-U1541C-4H,6W,43-44	30.7	1082.3	0.512291	-6.78	0.15	0.28	0.28	FD	PSU
383-U1541C-4H,6W,43-44*	30.7	1082.3	0.512294	-6.72	0.09	0.23	0.23	FD	PSU
383-U1541C-4H,6W,58-59	30.9	1092.4	0.512293	-6.74	0.11	0.20	0.20	FD	PSU
383-U1541C-4H,6W,73-74	31.0	1101.7	0.512299	-6.62	0.16	0.31	0.31	FD	PSU
383-U1541C-4H,6W,86-88	31.1	1114.2	0.512303	-6.54	0.18	0.28	0.28	Foraminifera	PSU
383-U1541C-4H,6W,113-114	31.4	1128.6	0.512299	-6.61	0.13	0.23	0.23	FD	PSU
383-U1541C-4H,6W,118-119	31.5	1130.9	0.512254	-7.49	0.13	0.34	0.34	Foraminifera	LDEO
383-U1541C-4H,6W,136-138	31.6	1139.5	0.512259	-7.40	0.21	0.26	0.26	Foraminifera	LDEO
383-U1541C-4H,6W,136-138*	31.6	1139.5	0.512267	-7.24	0.13	0.28	0.28	Foraminifera	PSU
383-U1541C-4H,6W,148-149	31.8	1144.8	0.512299	-6.62	0.11	0.20	0.20	FD	PSU
383-U1541C-5H,1W,10-11	32.9	1195.9	0.512335	-5.91	0.18	0.23	0.23	FD	PSU
383-U1541C-5H,1W,38-40	33.1	1204.6	0.512298	-6.63	0.22	0.27	0.27	Foraminifera	LDEO
383-U1541C-5H,1W,88-90	33.6	1211.7	0.512321	-6.19	0.12	0.34	0.34	Foraminifera	LDEO
383-U1541C-5H,1W,130-131	34.1	1217.5	0.512306	-6.48	0.11	0.20	0.20	FD	PSU
383-U1541C-5H,1W,138-140	34.1	1219.5	0.512302	-6.56	0.14	0.31	0.31	FD	PSU
383-U1541C-5H,2W,38-40	34.6	1231.1	0.512279	-7.01	0.13	0.31	0.31	FD	PSU
383-U1541C-5H,2W,38-40*	34.6	1231.1	0.512292	-6.76	0.12	0.23	0.23	Foraminifera	PSU
383-U1541C-5H,2W,40-41	34.7	1231.5	0.512266	-7.26	0.13	0.20	0.20	FD	PSU
383-U1541C-5H,2W,70-71	35.1	1243.2	0.512231	-7.93	0.25	0.51	0.51	Foraminifera	LDEO
383-U1541C-5H,2W,88-90	35.1	1245.8	0.512347	-5.67	0.17	0.31	0.31	FD	PSU
383-U1541C-5H,2W,90-91	35.2	1246.4	0.512341	-5.80	0.12	0.20	0.20	FD	PSU
383-U1541C-5H,2W,138-140	35.6	1259.4	0.512324	-6.12	0.12	0.23	0.23	FD	PSU
383-U1541C-5H,3W,15-16	35.9	1265.5	0.512299	-6.61	0.15	0.20	0.20	FD	PSU
383-U1541C-5H,3W,38-40	36.1	1271.5	0.512287	-6.84	0.15	0.31	0.31	FD	PSU
383-U1541C-5H,3W,88-90	36.6	1285.5	0.512323	-6.14	0.10	0.23	0.23	Foraminifera	PSU

383-U1541C-5H,3W,95-96	36.7	1287.6	0.512073	-6.07	0.11	0.20	0.20	FD	PSU
383-U1541C-5H,3W,138-140	37.1	1300.8	0.512307	-6.45	0.15	0.31	0.31	FD	PSU
383-U1541C-5H,4W,38-40	37.6	1324.2	0.512300	-6.59	0.12	0.28	0.28	Foraminifera	PSU
383-U1541C-5H,4W,50-51	37.8	1328.0	0.512315	-6.31	0.13	0.20	0.20	FD	PSU
383-U1541C-5H,5W,27-29	38.6	1352.3	0.512274	-7.10	0.15	0.28	0.28	FD	PSU
383-U1541B-5H,1W,95-96	39.1	1370.7	0.512321	-6.18	0.16	0.28	0.28	FD	PSU
383-U1541B-5H,1W,143-144	39.5	1382.4	0.512281	-6.97	0.10	0.23	0.23	FD	PSU
383-U1541B-5H,1W,145-146	39.6	1386.8	0.512256	-7.46	0.16	0.34	0.34	Foraminifera	LDEO
383-U1541B-5H,2W,30-31	39.9	1399.2	0.512216	-8.24	0.20	0.27	0.27	Foraminifera	LDEO
383-U1541B-5H,2W,53-55	40.1	1407.3	0.512294	-6.71	0.08	0.31	0.31	FD	PSU
383-U1541B-5H,2W,70-71	40.3	1412.5	0.512250	-7.57	0.11	0.20	0.20	FD	PSU
383-U1541B-5H,2W,120-121	40.8	1428.4	0.512275	-7.08	0.09	0.20	0.20	FD	PSU
383-U1541B-5H,3W,3-5	41.1	1440.0	0.512274	-7.10	0.13	0.31	0.31	Foraminifera	PSU
383-U1541B-5H,3W,20-21	41.3	1447.9	0.512255	-7.48	0.15	0.34	0.34	FD	LDEO
383-U1541B-5H,3W,30-31	41.4	1452.6	0.512302	-6.56	0.10	0.20	0.20	FD	PSU
383-U1541B-5H,3W,70-71	41.8	1472.1	0.512250	-7.58	0.10	0.34	0.34	Foraminifera	LDEO
383-U1541B-5H,3W,70-71*	41.8	1472.1	0.512274	-7.11	0.13	0.20	0.20	FD	PSU
383-U1541B-5H,3W,80-81	41.9	1478.4	0.512240	-7.77	0.14	0.34	0.34	FD	LDEO
383-U1541B-5H,3W,103-105	42.1	1489.0	0.512282	-6.94	0.12	0.25	0.25	FD	PSU
383-U1541B-5H,3W,110-111	42.2	1491.6	0.512272	-7.14	0.12	0.34	0.34	FD	LDEO
383-U1541B-5H,3W,120-121	42.3	1495.2	0.512320	-6.20	0.11	0.20	0.20	FD	PSU

1= Core Composite Depth Below Sea Floor (CCSF) measured in meter. The reported depths are the mid-point of the depth range of the sample.

2= Highest error is plotted. Internal and external errors are expressed in standard error and standard deviation respectively.

3= Marine archive used to generate the data. FD= Fish debris (includes both fossil fish teeth and broken bone fragments).

4= Lab names indicating where samples were analyzed. Pennsylvania State University (PSU), Lamont Doherty Earth Observatory (LDEO).

* Indicates replicate analyses.

REFERENCES

1. L. E. Lisiecki, M. E. Raymo, A Pliocene-Pleistocene stack of 57 globally distributed benthic δ^{18}O records. *Paleoceanography* **20**, PA1003 (2005).

2. N. G. Pisias, T. C. Moore, The evolution of Pleistocene climate: A time series approach. *Earth Planet. Sci. Lett.* **52**, 450–458 (1981).

3. T. D. Herbert, The mid-pleistocene climate transition. *Annu. Rev. Earth Planet. Sci.* **51**, 389–418 (2023).

4. P. U. Clark, D. Pollard, Origin of the middle pleistocene transition by ice sheet erosion of regolith. *Paleoceanography* **13**, 1–9 (1998).

5. H. Elderfield, P. Ferretti, M. Greaves, S. Crowhurst, I. N. McCave, D. Hodell, A. M. Piotrowski, Evolution of ocean temperature and ice volume through the mid-pleistocene climate transition. *Science* **337**, 704–709 (2012).

6. L. D. Pena, S. L. Goldstein, Thermohaline circulation crisis and impacts during the mid-Pleistocene transition. *Science* **345**, 318–322 (2014).

7. J. R. Farmer, B. Hönisch, L. L. Haynes, D. Kroon, S. Jung, H. L. Ford, M. E. Raymo, M. Jaume-Seguí, D. B. Bell, S. L. Goldstein, L. D. Pena, M. Yehudai, J. Kim, Deep Atlantic Ocean carbon storage and the rise of 100,000-year glacial cycles. *Nat. Geosci.* **12**, 355–360 (2019).

8. M. Yehudai, J. Kim, L. D. Pena, M. Jaume-Seguí, K. P. Knudson, L. Bolge, A. Malinverno, T. Bickert, S. L. Goldstein, Evidence for a Northern Hemispheric trigger of the 100,000-y glacial cyclicity. *Proc. Natl. Acad. Sci. U.S.A.* **118**, e2020260118 (2021).

9. T. B. Chalk, M. P. Hain, G. L. Foster, E. J. Rohling, P. F. Sexton, M. P. S. Badger, S. G. Cherry, A. P. Hasenfratz, G. H. Haug, S. L. Jaccard, A. Martínez-García, H. Pälike, R. D. Pancost, P. A. Wilson, Causes of ice age intensification across the Mid-Pleistocene transition. *Proc. Natl. Acad. Sci. U. S. A.* **114**, 13114–13119 (2017).

10. P. U. Clark, D. Archer, D. Pollard, J. D. Blum, J. A. Rial, V. Brovkin, A. C. Mix, N. G. Pisias, M. Roy, The middle Pleistocene transition: Characteristics, mechanisms, and implications for long-term changes in atmospheric pCO_2. *Quat. Sci. Rev.* **25**, 3150–3184 (2006).

11. S. K. V. Hines, C. D. Charles, A. Starr, S. L. Goldstein, S. R. Hemming, I. R. Hall, N. Lathika, M. Passacantando, L. Bolge, Revisiting the mid-Pleistocene transition ocean circulation crisis. *Science* **386**, 681–686 (2024).

12. M. Lambelet, T. van de Flierdt, K. Crocket, M. Rehkämper, K. Kreissig, B. Coles, M. J. A. Rijkenberg, L. J. A. Gerringa, H. J. W. de Baar, R. Steinfeldt, Neodymium isotopic composition and concentration in the western North Atlantic Ocean: Results from the GEOTRACES GA02 section. *Geochim. Cosmochim. Acta* **177**, 1–29 (2016).

13. A. Filippova, M. Frank, M. Kienast, J. Rickli, E. Hathorne, I. M. Yashayaev, K. Pahnke, Water mass circulation and weathering inputs in the Labrador Sea based on coupled Hf–Nd isotope compositions and rare earth element distributions. *Geochim. Cosmochim. Acta* **199**, 164–184 (2017).

14. H. Fröllje, K. Pahnke, B. Schnetger, H.-J. Brumsack, H. Dulai, J. N. Fitzsimmons, Hawaiian imprint on dissolved Nd and Ra isotopes and rare earth elements in the central North Pacific: Local survey and seasonal variability. *Geochim. Cosmochim. Acta* **189**, 110–131 (2016).

15. M. Kawabe, S. Fujio, Pacific ocean circulation based on observation. *J. Oceanogr.* **66**, 389–403 (2010).

16. C. Basak, K. Pahnke, M. Frank, F. Lamy, R. Gersonde, Neodymium isotopic characterization of Ross Sea Bottom Water and its advection through the southern South Pacific. *Earth Planet. Sci. Lett.* **419**, 211–221 (2015).

17. T. J. Williams, A. M. Piotrowski, J. N. W. Howe, C.-D. Hillenbrand, C. S. Allen, J. A. Clegg, The role of ocean circulation and regolith removal in triggering the Mid-Pleistocene Transition: Insights from authigenic Nd isotopes. *Quat. Sci. Rev.* **345**, 109055 (2024).

18. S. Robinson, R. Ivanovic, T. van de Flierdt, C. L. Blanchet, K. Tachikawa, E. E. Martin, C. P. Cook, T. Williams, L. Gregoire, Y. Plancherel, C. Jeandel, T. Arsouze, Global continental and marine detrital εNd: An updated compilation for use in understanding marine Nd cycling. *Chem. Geol.* **567**, 120119 (2021).

19. D. A. Hodell, K. A. Venz, C. D. Charles, U. S. Ninnemann, Pleistocene vertical carbon isotope and carbonate gradients in the South Atlantic sector of the Southern Ocean. *Geochem. Geophys. Geosyst.* **4**, 1–19 (2003).

20. N. J. Burls, A. V. Fedorov, D. M. Sigman, S. L. Jaccard, R. Tiedemann, G. H. Haug, Active Pacific meridional overturning circulation (PMOC) during the warm Pliocene. *Sci. Adv.* **3**, e1700156 (2017).

21. F. Pöppelmeier, M. Gutjahr, P. Blaser, H. Schulz, F. Süfke, J. Lippold, Stable Atlantic deep water mass sourcing on glacial-interglacial timescales. *Geophys. Res. Lett.* **48**, e2021GL092722 (2021).

22. G. L. Foster, D. Vance, Negligible glacial–interglacial variation in continental chemical weathering rates. *Nature* **444**, 918–921 (2006).

23. J. D. Blum, Y. Erel, "Sm-Nd isotope systematics," in *Treatise on Geochemistry*, H. D. Holland, K. K. Turekian, Eds. (Pergamon, 2003), pp. 365–392.

24. K. M. Jones, S. P. Khatiwala, S. L. Goldstein, S. R. Hemming, T. van de Flierdt, Modeling the distribution of Nd isotopes in the oceans using an ocean general circulation model. *Earth Planet. Sci. Lett.* **272**, 610–619 (2008).

25. B. Öhlander, J. Ingri, M. Land, H. Schöberg, Change of Sm-Nd isotope composition during weathering of till. *Geochim. Cosmochim. Acta* **64**, 813–820 (2000).

26. S. Sosdian, Y. Rosenthal, Deep-sea temperature and ice volume changes across the pliocene-pleistocene climate transitions. *Science* **325**, 306–310 (2009).

27. H. L. Ford, S. M. Sosdian, Y. Rosenthal, M. E. Raymo, Gradual and abrupt changes during the Mid-Pleistocene transition. *Quat. Sci. Rev.* **148**, 222–233 (2016).

28. C. E. Jasper, B. Dyer, B. T. Reilly, T. Williams, S. Hemming, M. E. Raymo, A 3.3-million-year record of Antarctic iceberg rafted debris and ice sheet evolution quantified by machine learning. *Paleoceanogr. Paleoclimatol.* **39**, e2024PA004897 (2024).

29. A. Starr, I. R. Hall, S. Barker, T. Rackow, X. Zhang, S. R. Hemming, H. J. L. van der Lubbe, G. Knorr, M. A. Berke, G. R. Bigg, A. Cartagena-Sierra, F. J. Jiménez-Espejo, X. Gong, J. Gruetzner, N. Lathika, L. J. LeVay, R. S. Robinson, M. Ziegler, L. Brentegani, T. Caley, C. D. Charles, J. J. Coenen, J. G. Crespin, A. M. Franzese, X. Han, S. K. V. Hines, F. J. Jimenez Espejo, J. Just, A. Koutsodendris, K. Kubota, R. D. Norris, T. P. dos Santos, J. M. Rolison, M. H. Simon, D. Tangunan, H. J. L. van der Lubbe, M. Yamane, H. Zhang, Expedition 361 Science Party, Antarctic icebergs reorganize ocean circulation during Pleistocene glacials. *Nature* **589**, 236–241 (2021).

30. Z. An, W. Zhou, Z. Zhang, X. Zhang, Z. Liu, Y. Sun, S. C. Clemens, L. Wu, J. Zhao, Z. Shi, X. Ma, H. Yan, G. Li, Y. Cai, J. Yu, Y. Sun, S. Li, Y. Zhang, C. Stepanek, G. Lohmann, G. Dong, H. Cheng, Y. Liu, Z. Jin, T. Li, Y. Hao, J. Lei, W. Cai, Mid-Pleistocene climate transition triggered by Antarctic Ice Sheet growth. *Science* **385**, 560–565 (2024).

31. M. O. Patterson, R. McKay, T. Naish, C. Escutia, F. J. Jimenez-Espejo, M. E. Raymo, S. R. Meyers, L. Tauxe, H. Brinkhuis, A. Klaus, A. Fehr, J. A. P. Bendle, P. K. Bijl, S. M. Bohaty, S. A. Carr, R. B. Dunbar, J. A. Flores, J. J. Gonzalez, T. G. Hayden, M. Iwai, K. Katsuki, G. S. Kong, M. Nakai, M. P. Olney, S. Passchier, S. F. Pekar, J. Pross, C. R. Riesselman, U. Röhl, T. Sakai, P. K. Shrivastava, C. E. Stickley, S. Sugasaki, S. Tuo, T. van de Flierdt, K. Welsh, T. Williams, M. Yamane, IODP Expedition 318 scientists, Orbital forcing of the East Antarctic ice sheet during the Pliocene and Early Pleistocene. *Nat. Geosci.* **7**, 841–847 (2014).

32. S. Rahmstorf, Is the Atlantic oveturning circulation approaching a tipping point? *Oceanography* **37**, 16–29 (2024).

33. J. C. H. Chiang, C. M. Bitz, Influence of high latitude ice cover on the marine Intertropical Convergence Zone. *Clim. Dyn.* **25**, 477–496 (2005).

34. J. R. Toggweiler, J. L. Russell, S. R. Carson, Midlatitude westerlies, atmospheric CO_2, and climate change during the ice ages. *Paleoceanography* **21**, PA2005 (2006).

35. A. P. Hasenfratz, S. L. Jaccard, A. Martínez-García, D. M. Sigman, D. A. Hodell, D. Vance, S. M. Bernasconi, H. F. Kleiven, F. A. Haumann, G. H. Haug, The residence time of Southern Ocean surface waters and the 100,000-year ice age cycle. *Science* **363**, 1080–1084 (2019).

36. Y. Watanabe, A. Abe-Ouchi, F. Saito, K. Kino, R. O'ishi, T. Ito, K. Kawamura, W.-L. Chan, Astronomical forcing shaped the timing of early Pleistocene glacial cycles. *Commun. Earth Environ.* **4**, 113 (2023).

37. N. C. Thomas, H. J. Bradbury, D. A. Hodell, Changes in North Atlantic deep-water oxygenation across the Middle Pleistocene Transition. *Science* **377**, 654–659 (2022).

38. F. Lamy, G. Winckler, C. A. A. Zarikian, I. S. Party, "Site 1541," in *Proceedings of the International Ocean Discovery Program* (2021).

39. J. L. Middleton, J. Gottschalk, G. Winckler, J. Hanley, C. Knudson, J. R. Farmer, F. Lamy, L. E. Lisiecki, Expedition 383 scientists, Evaluating manual versus automated benthic foraminiferal $\delta^{18}O$ alignment techniques for developing chronostratigraphies in marine sediment records. *Geochronology* **6**, 125–145 (2024).

40. L. Lin, D. Khider, L. E. Lisiecki, C. E. Lawrence, Probabilistic sequence alignment of stratigraphic records. *Paleoceanography* **29**, 976–989 (2014).

41. S. Ahn, D. Khider, L. E. Lisiecki, C. E. Lawrence, A probabilistic Pliocene–Pleistocene stack of benthic δ18O using a profile hidden Markov model. *Dyn. Stat. Clim. Syst.* **2**, dzx002 (2017).

42. T. Tanaka, S. Togashi, H. Kamioka, H. Amakawa, H. Kagami, T. Hamamoto, M. Yuhara, Y. Orihashi, S. Yoneda, H. Shimizu, T. Kunimaru, K. Takahashi, T. Yanagi, T. Nakano, H. Fujimaki, R. Shinjo, Y. Asahara, M. Tanimizu, C. Dragusanu, JNdi-1: A neodymium isotopic reference in consistency with LaJolla neodymium. *Chem. Geol.* **168**, 279–281 (2000).

43. J. Kim, S. L. Goldstein, L. D. Pena, M. Jaume-Seguí, K. P. Knudson, M. Yehudai, L. Bolge, North Atlantic deep water during pleistocene interglacials and glacials. *Quat. Sci. Rev.* **269**, 107146 (2021).

44. Y. Yan, M. L. Bender, E. J. Brook, H. M. Clifford, P. C. Kemeny, A. V. Kurbatov, S. Mackay, P. A. Mayewski, J. Ng, J. P. Severinghaus, J. A. Higgins, Two-million-year-old snapshots of atmospheric gases from Antarctic ice. *Nature* **574**, 663–666 (2019).

45. B. Bereiter, S. Eggleston, J. Schmitt, C. Nehrbass-Ahles, T. F. Stocker, H. Fischer, S. Kipfstuhl, J. Chappellaz, Revision of the EPICA Dome C CO_2 record from 800 to 600 kyr before present. *Geophys. Res. Lett.* **42**, 542–549 (2015).

46. B. Hönisch, N. G. Hemming, D. Archer, M. Siddall, J. F. McManus, Atmospheric carbon dioxide concentration across the mid-pleistocene transition. *Science* **324**, 1551–1554 (2009).

47. K. A. Dyez, B. Hönisch, G. A. Schmidt, Early Pleistocene obliquity-scale pCO_2 variability at ~1.5 million years ago. *Paleoceanogr. Paleoclimatol.* **33**, 1270–1291 (2018).

48. S. B. Jacobsen, G. J. Wasserburg, Sm-Nd isotopic evolution of chondrites. *Earth Planet. Sci. Lett.* **50**, 139–155 (1980).

49. S. L. Goldstein, S. R. Hemming, "Treatise on Geochemistry: The Oceans and Marine Geochemistry," in *Treatise on Geochemistry*, H. D. Holland, K. K. Turekian, H. Elderfield, Eds. (Elsevier, 2003), vol. 6, pp. 453.

50. C. Jeandel, T. Arsouze, F. Lacan, P. Techine, J. C. Dutay, Isotopic Nd compositions and concentrations of the lithogenic inputs into the ocean: A compilation, with an emphasis on the margins. *Chem. Geol.* **239**, 156–164 (2007).

51. T. Stichel, M. Frank, J. Rickli, B. A. Haley, The hafnium and neodymium isotope composition of seawater in the Atlantic sector of the Southern Ocean. *Earth Planet. Sci. Lett.* **317**, 282–294 (2012).

52. Y. Wu, L. D. Pena, R. F. Anderson, A. E. Hartman, L. L. Bolge, C. Basak, J. Kim, M. J. A. Rijkenberg, H. J. W. de Baar, S. L. Goldstein, Assessing neodymium isotopes as an ocean circulation tracer in the Southwest Atlantic. *Earth Planet. Sci. Lett.* **599**, 117846 (2022).

53. P. Carter, D. Vance, C. D. Hillenbrand, J. A. Smith, D. R. Shoosmith, The neodymium isotopic composition of waters masses in the eastern Pacific sector of the Southern Ocean. *Geochim. Cosmochim. Acta* **79**, 41–59 (2012).

54. C. Jeandel, H. Delattre, M. Grenier, C. Pradoux, F. Lacan, Rare earth element concentrations and Nd isotopes in the Southeast Pacific Ocean. *Geochem. Geophys. Geosyst.* **14**, 328–341 (2013).

55. C. Basak, Y. Wu, B. A. Haley, J. Muratli, L. D. Pena, L. Bolge, J. N. Fitzsimmons, R. M. Sherrell, S. L. Goldstein, Suspended particulate matter influence on dissolved Nd concentration and isotopic composition along GEOTRACES section GP16. *Earth Planet. Sci. Lett.* **635**, 118692 (2024).

56. J. A. Resing, P. N. Sedwick, C. R. German, W. J. Jenkins, J. W. Moffett, B. M. Sohst, A. Tagliabue, Basin-scale transport of hydrothermal dissolved metals across the South Pacific Ocean. *Nature* **523**, 200–203 (2015).

57. T. Stichel, K. Pahnke, B. Duggan, S. L. Goldstein, A. E. Hartman, R. Paffrath, H. D. Scher, T. A. G. Plume, Revisiting the hydrothermal neodymium contribution to seawater. *Front. Mar. Sci.* **5**, 96 (2018).

58. D. J. Piepgras, G. J. Wasserburg, Strontium and neodymium isotopes in hot springs on the East Pacific rise and guaymas basin. *Earth Planet. Sci. Lett.* **72**, 341–356 (1985).

59. A. N. Halliday, J. P. Davidson, P. Holden, R. M. Owen, A. M. Olivarez, Metalliferous sediments and the scavenging residence time of Nd near hydrothermal vents. *Geophys. Res. Lett.* **19**, 761–764 (1992).

60. K. Tachikawa, T. Arsouze, G. Bayon, A. Bory, C. Colin, J.-C. Dutay, N. Frank, X. Giraud, A. T. Gourlan, C. Jeandel, F. Lacan, L. Meynadier, P. Montagna, A. M. Piotrowski, Y.

Plancherel, E. Pucéat, M. Roy-Barman, C. Waelbroeck, The large-scale evolution of neodymium isotopic composition in the global modern and Holocene ocean revealed from seawater and archive data. *Chem. Geol.* **457**, 131–148 (2017).

61. T. Arsouze, J. C. Dutay, F. Lacan, C. Jeandel, Reconstructing the Nd oceanic cycle using a coupled dynamical–Biogeochemical model. *Biogeosciences* **6**, 2829–2846 (2009).

62. R. L. Rutberg, S. R. Hemming, S. L. Goldstein, Reduced North Atlantic deep water flux to the glacial Southern Ocean inferred from neodymium isotope ratios. *Nature* **405**, 935–938 (2000).

63. A. M. Piotrowski, S. L. Goldstein, S. R. Hemming, R. G. Fairbanks, Intensification and variability of ocean thermohaline circulation through the last deglaciation. *Earth Planet. Sci. Lett.* **225**, 205–220 (2004).

64. E. E. Martin, B. A. Haley, Fossil fish teeth as proxies for seawater Sr and Nd isotopes. *Geochim. Cosmochim. Acta* **64**, 835–847 (2000).

65. B. A. Haley, J. Du, A. N. Abbott, J. McManus, The impact of benthic processes on rare earth element and neodymium isotope distributions in the oceans. *Front. Mar. Sci.* **4**, 426 (2017).

66. J. Du, B. A. Haley, A. C. Mix, Evolution of the global overturning circulation since the last glacial maximum based on marine authigenic neodymium isotopes. *Quat. Sci. Rev.* **241**, 106396 (2020).

67. A. N. Abbott, B. A. Haley, J. McManus, Bottoms up: Sedimentary control of the deep North Pacific Ocean's εNd signature. *Geology* **43**, 1035–1035 (2015).

68. C. Basak, H. Fröllje, F. Lamy, R. Gersonde, V. Benz, R. F. Anderson, M. Molina-Kescher, K. Pahnke, Breakup of last glacial deep stratification in the South Pacific. *Science* **359**, 900–904 (2018).

69. M. Yehudai, L. E. Tweed, S. Ridge, Y. Wu, S. L. Goldstein, Effects of Past Nd seawater concentrations on Nd-isotope paleocirculation reconstructions: A bayesian approach. *Geophys. Res. Lett.* **50**, e2023GL104489 (2023).

70. T. van de Flierdt, A. M. Griffiths, M. Lambelet, S. H. Little, T. Stichel, D. J. Wilson, Neodymium in the oceans: A global database, a regional comparison and implications for palaeoceanographic research. *Philos. Trans. R. Soc. Math. Phys. Eng. Sci.* **374**, 20150293 (2016).

71. P. C. Tzedakis, M. Crucifix, T. Mitsui, E. W. Wolff, A simple rule to determine which insolation cycles lead to interglacials. *Nature* **542**, 427–432 (2017).

72. C. J. Berends, P. Köhler, L. J. Lourens, R. S. W. van de Wal, On the cause of the mid-pleistocene transition. *Rev. Geophys.* **59**, e2020RG000727 (2021).

73. J. N. W. Howe, A. M. Piotrowski, Atlantic deep water provenance decoupled from atmospheric CO_2 concentration during the lukewarm interglacials. *Nat. Commun.* **8**, 2003 (2017).
